# Structural and biochemical characteristics of citrus flowers associated with defence against a fungal pathogen

**DOI:** 10.1093/aobpla/plu090

**Published:** 2014-12-22

**Authors:** João Paulo Rodrigues Marques, Lilian Amorim, Geraldo José Silva-Junior, Marcel Bellato Spósito, Beatriz Appezzato-da Gloria

**Affiliations:** 1 Universidade de São Paulo, Escola Superior de Agricultura “Luiz de Queiroz”, Cx. Postal 9, CEP 13418-900, Piracicaba, SP, Brazil; 2 Fundo de Defesa da Citricultura – FUNDECITRUS, Av. Adhemar Pereira de Barros, 201 | CEP: 14807-040, Vila Melhado, Araraquara, SP, Brazil

**Keywords:** Antimicrobial activity, calcium oxalate crystals, citrus essential oils, *Citrus sinensis*, *Colletotrichum acutatum*, osmophores, plant anatomy, volatile organic compounds

## Abstract

The constitutive characters of plants can be structural or biochemical and play an important role in their defence against pathogens. Citrus postbloom fruit drop (PFD) caused by *Colletotrichum* spp. is one of the most important fungal diseases of citrus. The pathogen infects the flowers, leading to premature fruit drop and reducing citrus production. However, flower buds smaller than 8 mm long are usually not infected by *Colletotrichum* spp. Thus, this study investigated whether there are constitutive mechanisms in flower buds related to *Colletotrichum* spp. infection. We studied flower buds that were 2, 3, 4, 8, 12 and 15 mm long and petals, after anthesis, of sweet orange ‘Valência’ using light and scanning electron microscopy and histochemistry. We evaluated the effect of volatile organic compounds (VOCs) in flowers (R-limonene and linalool) on the *in vitro* growth of *Colletotrichum acutatum*. We found that the arrangement of the epidermal papillae in the petal primordia, the occurrence of prismatic crystals and the distribution of oil glands are the main differences between buds smaller than 8 mm and buds 8–15 mm long. Osmophores at the tips of petals produced and accumulated phenols, terpenes and lipophilic compounds. Flower buds smaller than 8 mm long have constitutive structural and biochemical barriers to *Colletotrichum* spp. infection. In addition, this is the first time that osmophores have been reported in citrus. Our study shows that natural terpenes of *Citrus* flowers inhibit the fungal growth *in vitro*, highlighting the potential use of terpenes for the chemical control of PFD in citrus.

## Abstract (precis)

The constitutive characters of plants can be structural or biochemical and play an important role in their defense against pathogens. We found that Citrus flower buds smaller than 8 mm long have constitutive structural and biochemical barriers to *Colletotrichum* spp. infection. In addition, this is the first time that osmophores are reported in *Citrus sinensis*. Our study shows that natural terpenes of Citrus flowers present a potential use for chemical control of *Colletotrichum* spp.

## Introduction

Plant species are attacked by a wide range of pathogens and herbivores, which can alter host survival, growth and reproduction (Wittstock and Gershenzon 2002; Agrios 2005). Plants defend themselves against these enemies using a combination of structural characters and biochemical reactions, which can be either constitutive or induced by attack (Wittstock and Gershenzon 2002; Agrios 2005; Hanley *et al*. 2007). Constitutive structural traits important to pathogen resistance include cell walls with different compositions, waxy epidermal cuticles, cuticles thickness, trichomes, idioblasts, sclereids and crystalliferous cells (Hudgins *et al*. 2003; Smith *et al*. 2006; Freeman and Beattie 2008; Koch and Ensikat 2008).

Citrus postbloom fruit drop (PFD) is a disease caused by *Colletotrichum acutatum* (Brown *et al*. 1996) and *C. gloeosporioides* (Lima *et al*. 2011; McGovern *et al*. 2012), both from phylum Ascomycota. The disease causes serious economic production losses of sweet oranges and is considered a limiting factor for producing countries (Timmer *et al*. 1994). In Brazil, losses caused by PFD can reach 80 % when the flowering coincides with periods of heavy rainfall in areas without the disease control (De Goes *et al*. 2008; Silva-Junior *et al*. 2014).

These pathogens can infect flower buds that are still closed (Fagan 1979), although most symptoms are observed during anthesis (Denham and Waller 1981). In the petals, the pathogen penetrates intra- and intercellularly, and also through the stomata. After invasion, acervuli (asexual fruiting bodies) are observed on both sides of the petals (Marques *et al*. 2013). Typical symptoms are orange-brown lesions (Fagan 1979). The lesions bearing salmon pink acervuli expand rapidly in favourable weather and soon involve all the petal tissues. This stage of the disease is often called blossom blight. The blighted petals remain firmly attached to the basal disc and become hard, dry and reddish brown (Denham and Waller 1981). In the stigma, small peach-brown to dark-brown necrotic spots are formed (Lin *et al*. 2001), but the pathogen does not penetrate through the epidermal cells of the stigma. A protective lipophilic layer rich in phenolic compounds is formed under the necrotic area, and crystals of calcium oxalate (CaO*_x_*) are produced where the pathogen is present (Marques *et al*. 2013). After infection, hormonal changes occur (Li *et al*. 2003; Lahey *et al*. 2004) leading to premature fruit drop and calyx retention for long periods (Timmer *et al*. 1994).

The developmental stages of flower buds are directly related to the infection caused by *C. acutatum* and efficiency of PFD control (Fagan 1979; Roberto and Borges 2001). Flower buds smaller than 8 mm are considered resistant to infection (Fagan 1979) and as the flower bud size increases, the buds become more susceptible to the infection. Thus, preventive applications of fungicides are recommended when most petals emerge above the calyx (Silva-Junior *et al*. 2014).

Citrus plants produce volatile organic compounds (VOCs) as secondary metabolites that play an important role in interaction routes with microorganisms. In citrus plants, the quantity of VOCs is variable and depends on the organ age and type. In the pericarp of green fruits, the oil is composed of β-pinene, sabinene and linalool; however, in ripe fruits, R-limonene and linalool become the most prevalent oils. In petals, the oils include terpenes R-limonene, myrcene, sabinine, linalool, terpineol and others (Attaway *et al*. 1966). To date, there is no evidence of the site where the synthesis and/or emission of volatiles occur on citrus petals. In several families, the sites where the synthesis occurs are called osmophores or scent glands (Vogel 1990; Dudareva and Pichersky 2000; Effemert *et al*. 2006). Osmophores have been reported only once for the Rutaceae (Bussell *et al*. 1995). Volatile organic compounds are produced naturally in plants and may be associated with constitutive biochemical defence mechanisms. They can also be extracted and used as an alternative to conventional chemical control, as they present fewer environmental hazards (Wightwick *et al*. 2010).

Floral buds smaller than 8 mm are considered resistant to pathogen infection (Fagan 1979). However, the mechanisms involved in this resistance have never been determined. We hypothesized that floral buds smaller than 8 mm have constitutive structural and chemical barriers that are associated with the resistance of those buds. This study aims to describe the anatomical structure of flower buds at different developmental stages to identify the presence of constitutive defences to infection caused by *C. acutatum*. We also evaluated the effect of the most common VOCs in flowers on the *in vitro* growth of *C. acutatum*, to verify their contribution to bud resistance.

## Methods

### Samples

Five-year-old potted sweet orange plants [*Citrus sinensis* var. Valência] were grown in 5-L plastic pots and maintained in a greenhouse at the Fund for Citrus Plant Protection (FUNDECITRUS), located in the municipality of Araraquara, São Paulo State, Brazil. Flowering was induced by pruning and water restriction. Small flower buds with 2, 3 and 4 mm, flower buds with expanded corolla (8, 12 and 15 mm), and petals after anthesis were collected for anatomical analyses.

### Light microscopy and histochemistry

The flower buds and the petals were collected, longitudinally sectioned and fixed in Karnovsky fixative (Karnovsky 1965; modified by phosphate buffer pH 7.2) for 48 h. During this phase, the samples were placed in a vacuum pump to remove air bubbles formed in the tissues. The samples were then dehydrated in a graded ethanol series and embedded in Leica historesin^®^ (Heraeus Kulzer, Hanau, Germany). Flower buds were also fixed in formalin-ferrous sulfate solution to detect phenolic compounds (Johansen 1940; Jensen 1962). The infiltration time of the flower buds was 1 month or more, depending on the developmental stage of the buds. The blocks were sliced in a Leica RM 2045 rotary microtome to produce 5–7-μm thick sections. The sections were mounted on glass slides and stained with 1 % Toluidine Blue (Sakai 1973) for histological analyses. The slides were mounted in synthetic resin Entellan^®^ (Merck, Darmstadt, Germany).

For histochemical analysis, Sudan black B was used to detect lipophilic compounds (Pearse 1968), 10 % ferric chloride was used to detect phenolic substances (Johansen 1940) and Ruthenium red for pectic compounds (Chamberlain 1932). After staining, the slides were embedded in the synthetic resin Entellan^®^. Calcium oxalate crystals were visualized under polarized light and their chemical nature was confirmed by analyzing their solubility in 1 % sulfuric acid (Johansen 1940). To confirm the presence of osmophores on the petal primordia and petal apex, samples were collected and immediately cross-sectioned on a sliding microtome Leica MS 2000R. To identify and characterize the osmophores' secretions the following histochemical techniques were used: rhodamine B (Jin *et al*. 2011) for sugar esters; Neutral Red (0.01 % in aqueous solution pH 8.0) to detect secretory activity (Dudareva and Pichersky 2000; Effemert *et al*. 2006); Nadi reagent for terpenoids (David and Carde 1964); Nile blue sulfate for acidic and neutral lipids (Cain 1947); and 10 % ferric chloride for phenolic compounds (Johansen 1940). Images from slides were captured digitally through a Leica DMLB microscope with a video camera attached to a PC, using Leica IM50 image analysis software.

### Scanning electron microscopy

Ten samples of each developmental stage of the flower buds and the petals were fixed in a Karnovsky solution adjusted to pH 7.2 using phosphate buffer (Karnovsky 1965, modified), dehydrated using a series of graded ethyl alcohols (10, 30, 50, 70, 90 and 100 %), critical point-dried using CO_2_ (Horridge and Tamm 1969), mounted on aluminium stubs using double-sided carbon tape and coated with a 30–40 nm gold film. Images were captured using a LEO VP 435 scanning electron microscope at an accelerating voltage of 20 kV.

### Effect of volatile organic compounds on the mycelial growth of *C. acutatum*

Volatile organic compounds of plants found in petals of sweet orange ‘Valência’ were tested *in vitro* for antifungal activity against *C. acutatum*. Two compounds were selected according to the study of Attaway *et al*. (1966): the R-limonene, 97 %, Sigma-Aldrich and linalool, 97 %, Sigma-Aldrich. R-limonene is one of the main components of essential citrus oils, the group of monoterpene hydrocarbons. Linalool is also found in essential oils from different citrus organs and belongs to the chemical group of monoterpene alcohols. Glass slides with doses of 1, 10, 100 and 1000 µg mL^−1^ of active ingredient of each compound were placed on one side of the polystyrene plate. On the other side of the plate, the culture medium BDA was added and a disc of mycelium of 0.5 cm diameter of *C. acutatum* (isolate Ca142 of Fundecitrus collection) was added to the medium. Plates containing water blades with and without the compounds served as control. The plates were sealed with Parafilm and maintained in growth chambers at 25 °C. After 7 days, the diameter of the colony was measured. Growth inhibition was calculated and the values were used to estimate the EC_50_ (effective concentration to reduce growth by 50 %) and minimal inhibitory concentration (MIC). The experiment was performed in triplicate with three replications; each plate was considered one repetition.

## Results

### Structural and histochemical analyses

**Figure 1.**
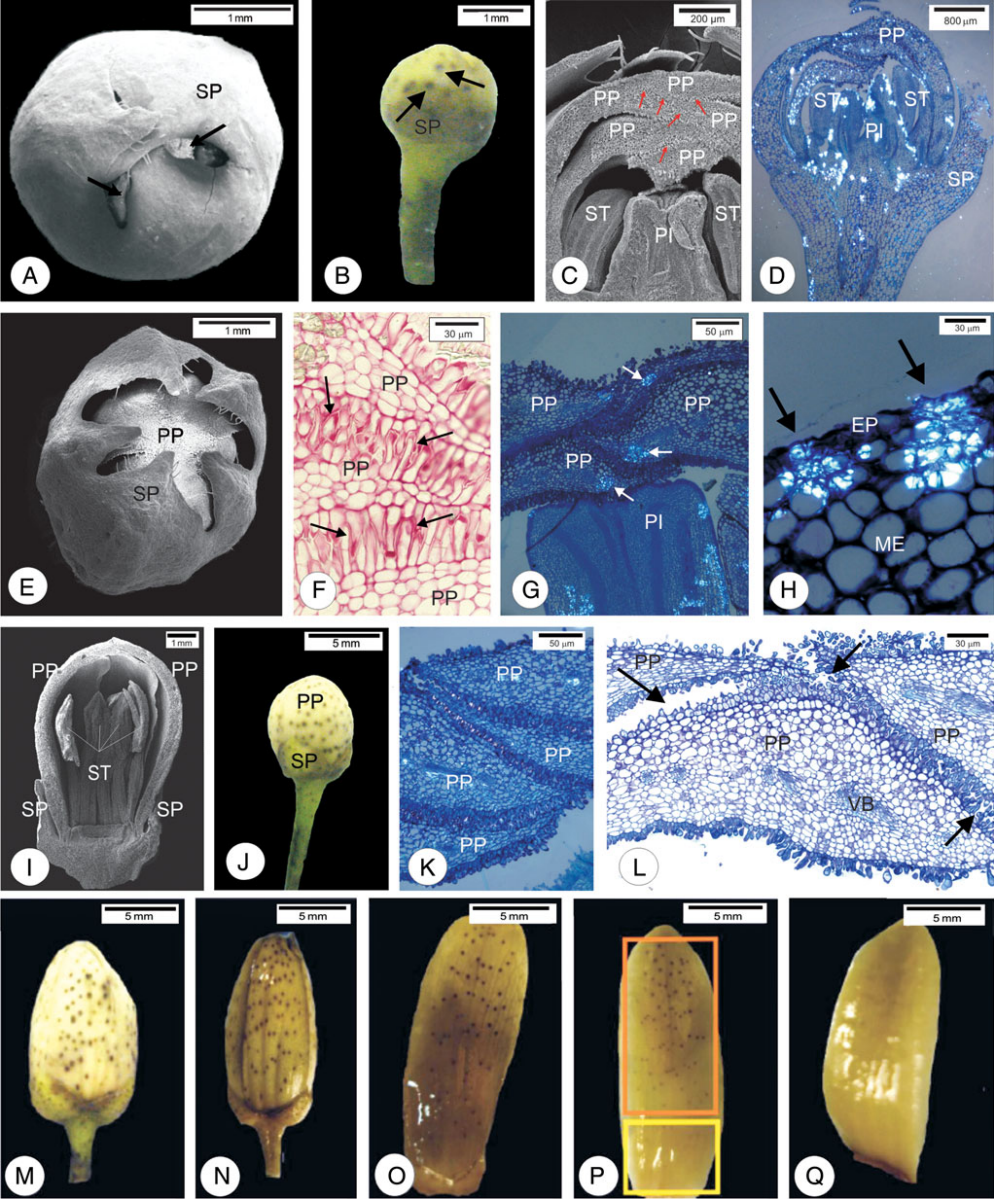
Scanning electron micrographs (A, C, E, I), photomicrographs under non-polarized light (F, L) and polarized light (D, G, H, K) and photographs (B, M–Q) of flower buds of sweet orange (*C. sinensis* ‘Valência’). (A–D) Flower buds 2 mm long. (A) Note the small exposure of the petal primordia (arrow). (B) Oil gland of the sepal primordia (arrows). (C) Apex of the petal primordia show compact pattern of intermixing (arrows). (D) Overview of the bud under polarized light. Note crystals distributed in the primordia. (E–H) Flower bud 4 mm long. (E) Note larger exposure of the corolla. (F) Arrangement in the apexes of the papillary cells of the petal primordia (arrows) after staining with ruthenium red. (G) Crystals grouped in the apexes of the petal primordia. (H) Crystals inside the cells surrounding the substomatal chambers of petal primordia. (I–K) Flower buds 8 mm long. (I and J) Note the exposure of the corolla. (K) Absence of crystals in the mesophyll cells of the petals. (L and M) Flower buds 12 mm long. (L) Detail of the apex of the flower bud where the loose arrangement of papillary cells is observed (arrows). (M–Q) Fixation with ferrous sulfate in formalin. (N) Flower buds 15 mm long. (O) Flower bud after anthesis. (P) Abaxial face of petal. Note the smaller number of oil glands along the petal base (yellow rectangle) when compared to the apex (orange rectangle). (Q) Note the absence of oil glands on the adaxial face of the petal. EP, epidermis; ST, stamens; ME, mesophyll; PI, pistil; PP, petal primordia; SP, sepal primordia; VB, vascular bundle.

**Figure 2.**
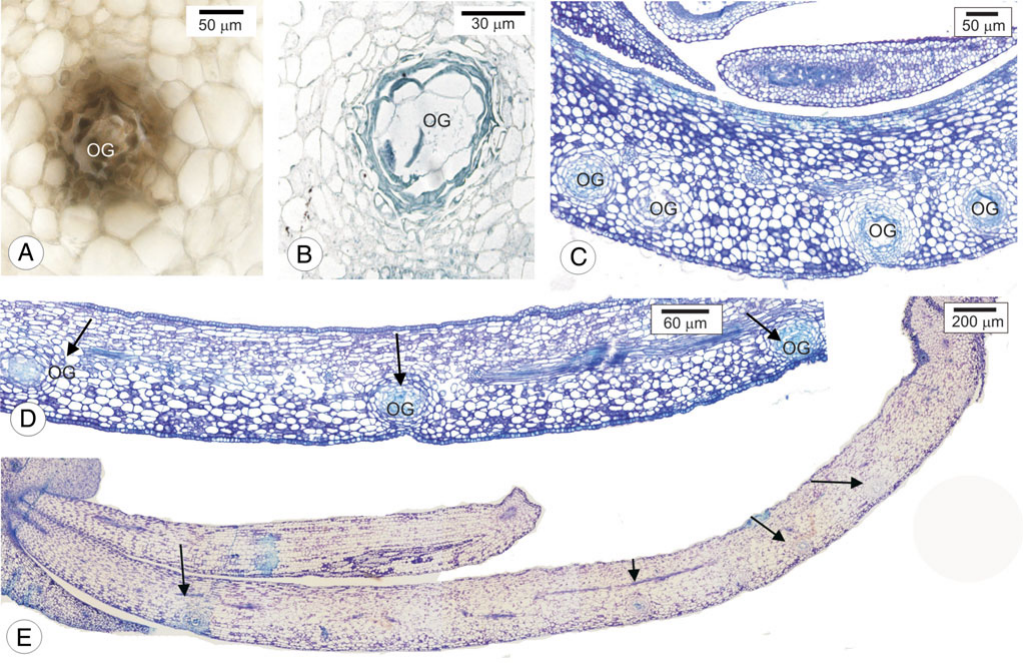
Cross-sections of oil glands in petal primordia of sweet orange (*Citrus sinensis* ‘Valência’). (A) Oil gland cells reacting positively to ferrous sulfate in formalin. (B) Oil gland cells stained with Sudan Black B. (C–E) Distribution of oil glands (arrows) in flower buds 4, 8 and 12 mm long. OG, oil gland.

In flower buds 2 mm long, the apexes of the five sepal primordia overlap (Fig. 1A and B), protecting the other flower whorls (Fig. 1C). In flower buds 3–4 mm long, sepal primordia separated to accommodate expansion of the underlying corolla (Fig. 1E). After fixation with ferrous sulfate in formalin, the sepal primordia have oil glands high in phenolic compounds (Figs 1B and 2A). Petal tips overlap and distal papillate regions press together, especially in 2, 3 and 4 mm buds (Fig. 1C, F and G), relative to the stages of 8–12 mm long (Fig. 1K and L).

Under polarized light, buds shorter than 8 mm have CaO*_x_* crystals in the mesophyll, either dispersed (Fig. 1D) or grouped (Fig. 1G). This grouping occurs near the apexes of the petal primordia. The crystals are also observed inside the cells surrounding substomatal chambers (Fig. 1H).

In the buds 8 mm long (Fig. 1I and J) or longer (Fig. 1M and N), petal primordia are exposed, distal papillate regions begin to separate (Fig. 1K and L) and few CaO*_x_* crystals are seen (Fig. 1K).

Flower buds up to 8 mm long have uniform distribution of oil glands (Fig. 1J). However, as the flower buds develop, oil glands nearer the petal base spread, whereas oil glands at petal tips remain clustered (Fig. 1M and N). This pattern continues in subsequent stages of flower development (Fig. 1O and P). The flower buds expose only the abaxial face where oil glands are present. Anthesis exposes the adaxial petal surface, which is free of oil glands (Fig. 1Q).

Oil glands contain phenolic (Fig. 2A) and lipophilic (Fig. 2B) compounds. In flower buds 4 mm long, glands are concentrated near each other (Fig. 2C), but become more spread out as petals enlarge in 8 mm- (Fig. 2D) and 12-mm-long buds (Fig. 2E). The distribution pattern of glands in buds 12 mm long is uneven, as most glands are observed near the apex of the petal primordia (Fig. 2E).

**Figure 3.**
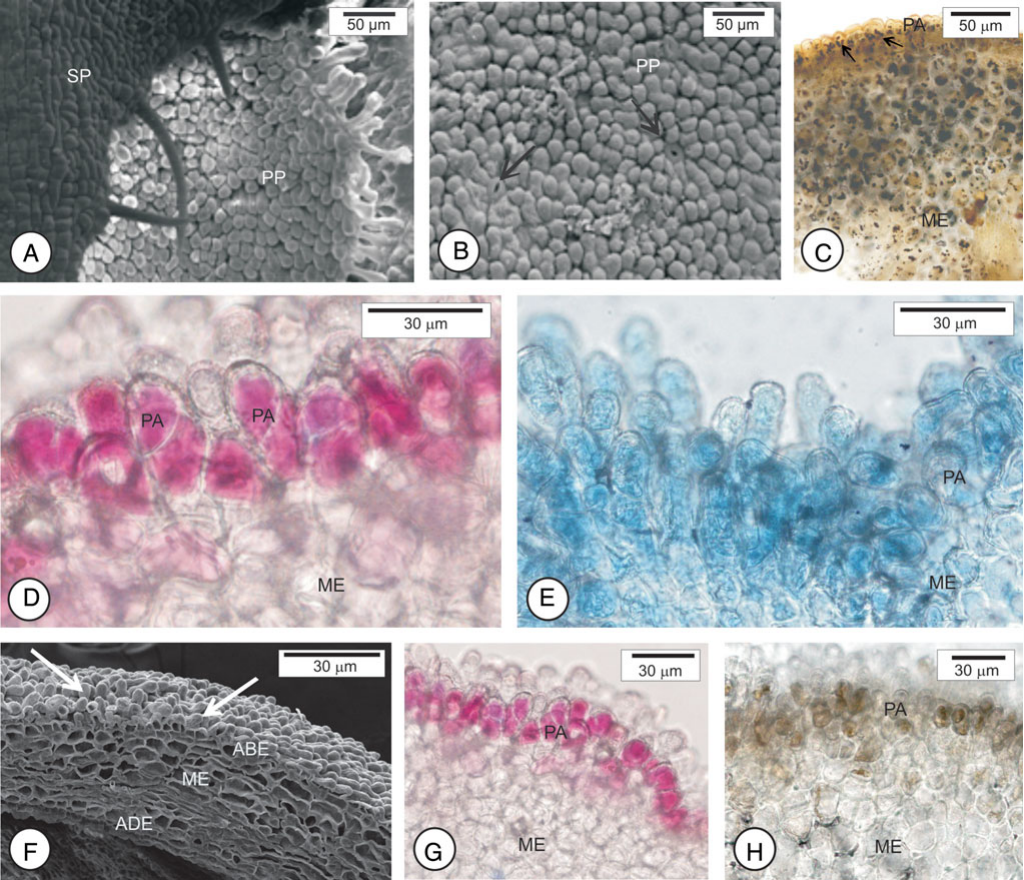
Scanning electron micrographs (A, B, F) and photomicrographs (C–E, G, H) of osmophores in different developmental stages of flower buds of sweet orange (*C. sinensis* ‘Valência’). (A–C) Flower buds 3 mm long. The region exposed is composed of papillary cells among which the stomata occur (arrow in B). (C) Note starch accumulation in the papillary cells. (D and E) Buds 8 mm long. Papillary cells stained with neutral red dye (D) and with Nile blue sulfate (E). (F–H) buds 12 mm long. (F) Papillary cells (arrows) that react positively to neutral red dye (G) and ferric chloride (H). ABE, abaxial epidermis; ADE, adaxial epidermis; ME, mesophyll; PA, papillae; PP, petal primordia; SP, sepal primordia.

The region of exposed corolla in flower buds 2–4 mm long is composed of papillary cells (Fig. 3A and B). In flower buds 2 mm long, starch accumulates in cells of the mesophyll and papillae (Fig. 3C). In the other stages analyzed, in buds 3, 4, 8 and 12 mm long (Fig. 3F), papillary cells react positively to the neutral red dye (Fig. 3D and G), to Nile blue sulfate (Fig. 3E) and to ferric chloride (Fig. 3H). These papillae also have a positive reaction to the NADI reagent, indicating the presence of terpenes (not shown).

**Figure 4.**
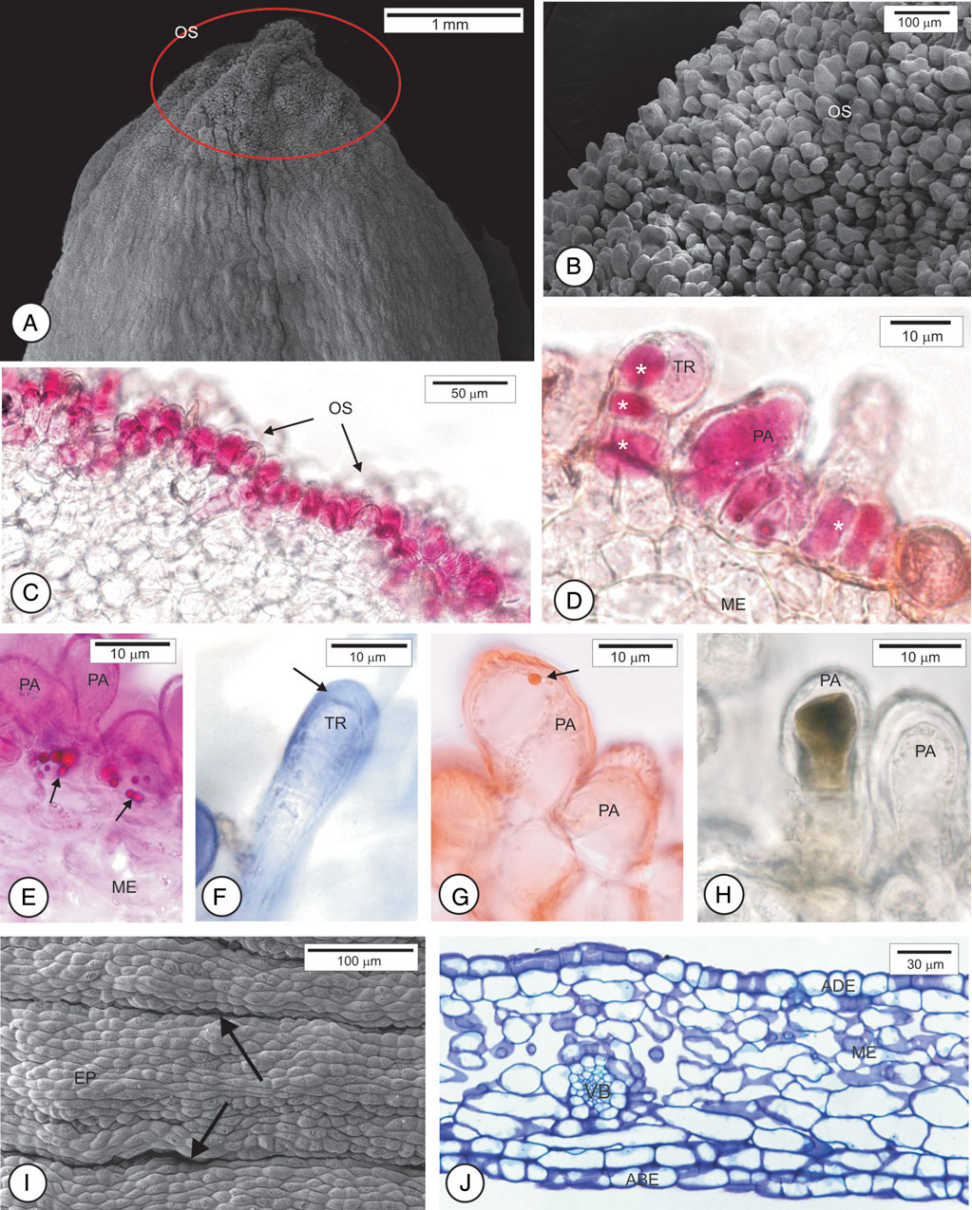
Scanning electron micrographs (A, B, H) and photomicrographs (C–G, I) of healthy petal of sweet orange (*C. sinensis* ‘Valência’). (A) Overview of the petal with the apex delimited by the circle. (B and C) Detail of the osmophore (OS) in the region delimited in A. (C and D) Papillae and trichome with contents stained with neutral red dye (*). (E) Sugar esters (arrows) evidenced by Rhodamine B. (F) Positive reaction to the NADI reagent at the edge of the trichome (arrow). (G) Lipid droplet stained with Sudan IV. (H) Phenolic content evidenced by ferric chloride. (I) Overview of medium region of the petal where depressions (arrows) and protrusions are observed. (J) Cross-section at the petal base. ABE, abaxial epidermis; ADE, adaxial epidermis; VB, vascular bundle; ME, mesophyll; PA, papillae; TR, trichome; EP, epidermis.

The petals from the fully open flowers show distinct epidermal features at the apex, medium and base. At the apex (Fig. 4A), cells are papillary (Fig. 4B) among which some uniseriate trichomes occur (Fig. 4C). The histochemical tests allowed us to confirm that the petal apex is an osmophore. Epidermal cells stained with neutral red dye (Fig. 4C and D), show sugar esters (Fig. 4E), and react positively to the NADI reagent, indicating the presence of terpenes (Fig. 4F) and lipid drops (Fig. 4G). Some cells react positively to ferric chloride indicating the presence of phenolic compounds (Fig. 4H). The surface of the medium region shows depressions and protrusions and the papillae are less prominent than at the apex and are covered with striated cuticle (Fig. 4I). At the petal base, epidermal cells are tabular (Fig. 4J) and the cuticle shows no stretch marks.

The mesophyll is homogeneous and composed of parenchyma braciform cells (Fig. 4J). In the mesophyll, oil glands are turned only to the abaxial surface (Fig. 2C–E). The vascular bundles are collateral (Fig. 4J). The stomata occur on both faces.

### Effect of VOCs on the mycelial growth of *C. acutatum*

**Figure 5.**
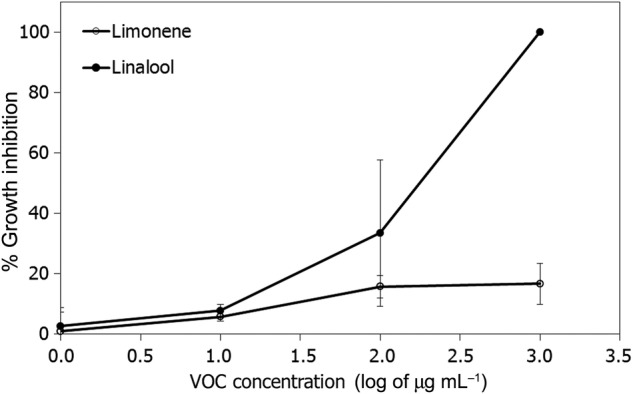
Inhibition of mycelial growth of *C. acutatum* at different concentrations of R-limonene and linalool. Dots represent the average of three experiments and three replicates for each rate.

The monoterpene alcohol linalool showed higher inhibition of the mycelial growth of *C. acutatum* than monoterpene hydrocarbon R-limonene (Fig. 5). Linalool reduced the mycelial growth of *C. acutatum* by 30 % at 100 µg mL^−1^, against 15 % inhibition of R-limonene at the same dose. At 1000 µg mL^−1^, linalool inhibited mycelial growth 100 %, while R-limonene remained <20 % inhibition. At lower concentration of some repetitions of R-limonene, small stimulation of the growth of the *C. acutatum* was observed. The EC_50_ and MIC for the linalool are between 100 and 1000 µL L^−1^; however, for the limonene, the EC_50_ is greater than 1000 µg mL^−1^ (Fig. 5).

## Discussion

The anatomical analyses of the flower buds show structural characteristics that may explain the lower incidence of infection in buds smaller than 8 mm. The compact arrangement of papillae in the petal primordia in young flower buds could be considered a structural barrier to infection caused by *C. acutatum*. On the other hand, buds longer than 8 mm show loose arrangement, thus offering less resistance to the infection caused by this pathogen. These results corroborate Fagan (1979) in Belize, where flower rot symptoms were expressed only in flower buds during the expansion stage of the corolla and only in those buds longer than 8 mm. The author states that flower buds smaller than 8 mm may be more resistant to infection; however, susceptibility increases dramatically as the flower buds age, remaining high until anthesis.

The lower infection rate of flower buds smaller than 8 mm caused by *C. acutatum* can also be associated with the presence of CaO*_x_* crystals in the petal primordia. According to Ceita *et al*. (2007), crystal degradation produces H_2_O_2_ in specific points in the tissue and plays an important role in programmed cell death, acting effectively in pathogen control. The application of Ca in large quantities leads to the formation of intracellular CaO*_x_* crystals (Choi *et al*. 2000; Faheed *et al*. 2013). There is a positive correlation between the Ca concentration provided to the plant and the number of crystals formed (Zindler-Frank *et al*. 2001), as well as with their length and width (Faheed *et al*. 2013). Therefore, *C. sinensis* plants receiving high doses of Ca possibly show a large number of CaO*_x_* crystals in flower buds and, consequently, are more resistant to infection caused by *C. acutatum*. Studies on nutrition based on different Ca sources could be conducted to further investigate this possibility.

The occurrence of osmophores at the petal apexes, consisting of papillary cells and glandular trichomes, has not yet been documented for *C. sinensis*. These osmophores are morphologically similar to those described in some Orchidaceae (Pridgeon and Stern 1985; Pansarin *et al*. 2009; Melo *et al*. 2010). In smaller flower buds, cells in the mesophyll of petal primordia accumulate starch in the early developmental stages of the osmophores. This accumulation may supply carbon for later production of VOCs (Pridgeon and Stern 1983; Stern *et al*. 1987; Effemert *et al*. 2006). According to Knudsen *et al*. (1993) the VOCs released by osmophores in 60 families of plants are: hydrocarbons, esters, ethers, aldehydes, ketones, terpenes, benzenoids, phenylpropanoids, isoprenoids and nitrogen- and sulfur-containing compounds. Among the terpenes, the mono- and sesquiterpenes stand out (Dudareva and Pichersky 2000). The osmophores of *C. sinensis* produce and accumulate phenols, terpenes and lipophilic compounds in their cells (Fig. 4). These substances have a recognized role as antifungal agents (Caccioni *et al*. 1998; Lattanzio *et al*. 2006). In addition to their ecological function (Proctor *et al*. 1996), the compounds accumulated by osmophore cells may act as a constitutive chemical barrier. Further studies need to be conducted to identify the compounds secreted by *C. sinensis* osmophores.

In flower buds smaller than 8 mm, oil glands are nearer to each other when compared with larger flower buds. The low incidence of lesions in flower buds smaller than 8 mm could possibly be linked to the concentrated oil glands, which may defend the tissues against pathogen attack. Essential oils of *C. sinensis* represent a constitutive defence against pathogens (Caccioni *et al*. 1998). Some terpenes in citrus oil glands have been shown to inhibit bacteria (Dabbah *et al*. 1970) and fungi (Moleyar and Narasimham 1986; Caccioni *et al*. 1998; Sharma and Tripathi 2008; Viuda-Martos *et al*. 2008).

Linalool is expressed in petals and in green and ripe fruit, while limonene appears mostly in ripe fruit. Linalool showed high antifungal activity against *C. acutatum* at doses higher than 100 µg mL^−1^; conversely, limonene showed low antifungal activity at 1000 µg mL^−1^. Linalool is usually the predominant VOC in flowers of different citrus species, mainly in sweet orange. However, there is a wide variation in concentration of this compound, from 22 to 52 % depending on the cultivar and the developmental stage of the flower (Alissandrakis *et al*. 2003; Jabalpurwala *et al*. 2009; Azam *et al*. 2013). In tangerines, limonene and myrcene did not inhibit *in vitro* germination of conidia of *Alternaria alternata* at the dose of 130 µg mL^−1^. However, linalool showed an antifungal effect, inhibiting >97 % of the germination of conidia by >97 % at a similar dose (Yamasaki *et al*. 2007). In addition, limonene showed a stimulating effect on the germination of *Penicillium digitatum* and *P. italicum*, causal agents of mould in citrus fruits, (Droby *et al*. 2008). The presence of alcoholic terpenes such as linalool in oil glands on petals of the Valência orange may be linked to the resistance of flower buds smaller than 8 mm. Nevertheless, our results suggest that the presence of limonene in flowers of *C. sinensis* is not associated with the mechanisms of resistance to infection caused by *C. acutatum.* Transformed orange plants that accumulated a smaller amount of limonene in the fruit peel have a higher resistance to fungus *P. digitatum* and bacterium *Xanthomonas citri* subsp. citri, causal agent of citrus canker (Rodriguez *et al*. 2013).

The high susceptibility of the fully open flower to infection caused by *C. acutatum* may be related to structural features of the petal flower after opening that promote exposure of the adaxial face of the petal (which has no oil glands). In addition, this surface is composed of depressions and protrusions, which may facilitate the deposition of conidia and the formation of appressoria that occur preferentially along the anticline walls of epidermal cells (Marques *et al*. 2013). Petals could be more vulnerable to fungal infection because of softened cell walls (due to auxin-induced cell expansion). Ultrastructural studies of the parenchyma cell wall may contribute to better understanding of the vulnerability of the parenchyma cell to fungal infection.

## Conclusions

We conclude that the difference in response to infection caused by the fungus *C. acutatum* in flower buds of *C. sinensis* at different developmental stages may be linked to constitutive structural and biochemical barriers. The presence of CaO*_x_* crystals, the pattern of papilla and the distribution, density and composition of citrus glands should be taken into consideration in further studies on disease control, once these constitutive natural defences are more evident in flower buds smaller than 8 mm in relation to flower buds longer than 8 mm, susceptible to the disease. For instance, spraying fungicides to control the disease can be delayed until the flower buds are longer than 8 mm. In addition, this study showed for the first time the site where synthesis and emission of volatiles occur in citrus petals. The site is characterized as an osmophore that produces and accumulates phenol, terpenes and lipophilic compounds. In addition, we demonstrate that alcoholic compounds such as monoterpenes present in citrus flowers have antifungal action against *C. acutatum* and may be associated with constitutive biochemical defences. These compounds have the potential to be used in further studies on chemical control of the pathogen or on genetic transformation and their overexpression in tissues.

## Sources of Funding

This work was supported by São Paulo Research Foundation (FAPESP—projects 2008/541764, 2009/00425-6).

## Contributions by the Authors

J.P.R.M. conceived of the project, planned the research, carried out the techniques and drafted the manuscript. L.A. and M.B.S. discussed the phytopathological aspects and contributed with the critical review of the manuscript. G.J.S.-J. carried out the *in vitro* experiments of antifungal effect of VOCs. B.A.-d.G. oversaw the research, conceived of the project, made the critical histological analysis and participated of the manuscript writing. All authors read and approved the final manuscript.

## Conflicts of Interest Statement

None declared.
